# Multisite assessment of the impact of cell-free DNA-based screening for rare autosomal aneuploidies on pregnancy management and outcomes

**DOI:** 10.3389/fgene.2022.975987

**Published:** 2022-08-29

**Authors:** Tamara Mossfield, Erica Soster, Melody Menezes, Gloudi Agenbag, Marie-Line Dubois, Jean Gekas, Tristan Hardy, Monika Jurkowska, Pascale Kleinfinger, Kelly Loggenberg, Pablo Marchili, Roberto Sirica, on behalf of the Global Expanded NIPT Consortium

**Affiliations:** ^1^ Genea, Sydney, NSW, Australia; ^2^ Labcorp Women’s Health and Genetics, Laboratory Corporation of America, San Diego, CA, United States; ^3^ Monash IVF Genetics, Monash IVF Group, Richmond, VIC, Australia; ^4^ Next Biosciences, Johannesburg, South Africa; ^5^ Faculty of Medicine, Laval University, Quebec City, QC, Canada; ^6^ CHU de Quebec Research and Mother and Child Center, Department of Medical Genetics, University Hospital of Quebec, Laval University, Quebec City, QC, Canada; ^7^ Genomed SA, Warsaw, Poland; ^8^ Laboratory CERBA, Saint-Ouen-l’Aumône, France; ^9^ Veragen, Buenos Aires, Argentina; ^10^ AMES, Centro Polidiagnostico Strumentale, Srl, Naples, Italy

**Keywords:** noninvasive prenatal testing, rare autosomal aneuploidy, pregnancy outcome, mosaicism, genetic counseling, genome-wide

## Abstract

Cell-free (cf) DNA screening is a noninvasive prenatal screening approach that is typically used to screen for common fetal trisomies, with optional screening for sex chromosomal aneuploidies and fetal sex. Genome-wide cfDNA screening can screen for a wide variety of additional anomalies, including rare autosomal aneuploidies (RAAs) and copy number variants. Here, we describe a multi-cohort, global retrospective study that looked at the clinical outcomes of cases with a high-risk cfDNA screening result for a RAA. Our study cohort included a total of 109 cases from five different sites, with diagnostic outcome information available for 68% (74/109) of patients. Based on confirmatory diagnostic testing, we found a concordance rate of 20.3% for presence of a RAA (15/74) in our study population. Pregnancy outcome was also available for 77% (84/109) of cases in our cohort. Many of the patients experienced adverse pregnancy outcomes, including intrauterine fetal demise, fetal growth restriction, and preterm birth. These adverse outcomes were observed both in patients with fetal or placental confirmation of the presence of a RAA, as well as patients that did not undergo fetal and/or placental diagnostic testing. In addition, we have proposed some suggestions for pregnancy management and counseling considerations for situations where a RAA is noted on a cfDNA screen. In conclusion, our study has shown that genome-wide cfDNA screening for the presence of rare autosomal aneuploidies can be beneficial for both patients and their healthcare practitioners. This can provide a possible explanation for an adverse pregnancy outcome or result in a change in pregnancy management, such as increased monitoring for adverse outcomes.

## Introduction

Clinical availability of cell-free (cf) DNA screening (also known as noninvasive prenatal testing [NIPT] or noninvasive prenatal screening [NIPS]) has resulted in a paradigm shift in chromosomal prenatal screening, with testing options quickly expanding from trisomy 21 screening only to the inclusion of screening for trisomies 13 and 18 (Nicolaides et al., 2012; Palomaki et al., 2012; Nicolaides et al., 2013), as well as optional screening for fetal sex and sex chromosomal aneuploidies (Mazloom et al., 2013; Samango-Sprouse et al., 2013). However, this cfDNA screening approach will still miss about 17% of clinically relevant fetal chromosomal abnormalities (Wellesley et al., 2012). In more recent years, the scope of cfDNA screening has broadened to encompass genome-wide screening for rare autosomal aneuploidies (RAAs) and partial deletions and duplications (i.e., copy number variants, including select microdeletion syndromes) as an option for some clinicians and their patients. Several studies have shown strong test performance for the detection of these additional anomalies by genome-wide cfDNA screening (Pescia et al., 2017; Pertile et al., 2021; Soster et al., 2021).

The screen-positive rate for RAAs in large genome-wide cfDNA screening studies has been shown to range from 0.12% (Scott et al., 2018) to 1.1% (Van Opstal et al., 2018). Benn *et al.* (Benn et al., 2019) pooled data from a number of studies and found that rare autosomal trisomies (RATs; i.e., an autosomal trisomy other than trisomy 21, 18, or 13) were present in 0.32% of cfDNA samples compared to 0.41% of trophoblast samples from chorionic villus sampling (CVS). Rare autosomal aneuploidies can be associated with a number of adverse pregnancy outcomes, including early miscarriage, intrauterine fetal demise (IUFD), fetal growth restriction (FGR), structural fetal anomalies, and preterm birth (Pertile et al., 2017; Eggenhuizen et al., 2021); as well as a proportion of cases in which there are no adverse pregnancy outcomes and birth of a healthy child. As cfDNA screening analyzes DNA released by apoptotic placental trophoblasts present in maternal plasma, a high-risk call for a RAA may be indicative of confined placental mosaicism (CPM) and not true fetal aneuploidy. A recent study (Van Opstal et al., 2020) found that cell-free DNA screening may be more sensitive than CVS for detection of CPM involving the cytotrophoblast. Although CPM cases are typically called as false positives for cfDNA screening, some studies have shown that CPM for autosomal aneuploidies can lead to adverse pregnancy outcomes, particularly for CPM involving chromosome 16 (Vaughan et al., 1994; Zimmermann et al., 1995; Sánchez et al., 1997; Van Opstal et al., 1998; Eggenhuizen et al., 2021), as well as chromosomes 2, 3, 7, 13, 15, and 22 (Eggenhuizen et al., 2021). In addition, a high-risk cfDNA result can be a marker for uniparental disomy (UPD, i.e., two copies of a whole chromosome derived from one parent (Benn, 2021)), especially if the CPM involves chromosomes that carry imprinted genes associated with defined syndromes (Mardy and Wapner, 2016; Grati et al., 2020).

With the increasing availability and uptake of genome-wide cfDNA screening, information on the clinical impact of rare autosomal aneuploidies will help guide pregnancy management and counseling. The majority of studies to date have had either small data sets or have not detailed the pregnancy and birth outcomes of patients in their study cohort. Here, we describe a global multi-site study that looked at the pregnancy and clinical outcomes following a high-risk RAA call in a large number of cases. We also wanted to determine if having a high-risk screening result for a rare autosomal aneuploidy was beneficial for management of the pregnancy in our cohort, and whether this was useful information for the healthcare provider and the pregnant patient. Results from our study, together with the experience of our Consortium members, were considered in an attempt to provide suggestions for pregnancy management and appropriate counselling of pregnant patients when these aneuploidies are detected.

## Materials and methods

### Study and patient details

All members of the Global Expanded NIPT Consortium were invited to submit details from their laboratory/clinic of cases reported as a rare autosomal aneuploidy following genome-wide cfDNA screening. Cases had to involve RAAs on a single whole chromosome only. All known cases from the time each site commenced genome-wide cfDNA screening, up to and including cases reported in 2021, were considered for inclusion in the study. The end date for cases reported in 2021 varied by contributing sites from May 2021 to October 2021, with one site not contributing any cases for 2021. The patient samples in this retrospective data analysis study were collected as part of routine cfDNA screening in either general or high-risk populations, dependent on the protocols and standards of care of each contributing site. The study included both singleton and twin pregnancies; however, not all sites performed genome-wide cfDNA screening for twin pregnancies. Referral indications for cfDNA screening were collected as noted on the Test Requisition Form (TRF).

Information on human chorionic gonadotropin levels, PAPP-A levels, inhibin levels, and nuchal translucency (NT) were available for some of the patients in our study cohort. These were collected either as part of conventional screening, as part of the first trimester anatomy scan, or as part of preeclampsia screening.

All data was de-identified before analysis was carried out. The study received an IRB exemption from WCBIRB as it does not meet the definition of human subject research as defined in 45 CFR 46.102; specifically, the research involves analysis of retrospectively collected de-identified data only.

### Genome-wide cfDNA screening

Genome-wide cfDNA screening and analysis was carried out at each site following site-specific routine laboratory procedures. All of the sites used a massively parallel whole-genome next-generation sequencing approach. Four of the five sites used the VeriSeq^TM^ NIPT Solution v2 assay (Illumina, Inc.) (Pertile et al., 2021); the other site used the TruSeq^TM^ Nano 16 sample protocol (Illumina, Inc.) for sequencing of the cfDNA (Illumina, 2017).

Where available, the fetal fraction (FF) was provided for each RAA case that was included in the study. A subset of cases at one of the sites had non-interpretable FF results. Presence of a RAA was thought to be the underlying etiology for interference with the bioinformatics analysis. In these cases, the result was considered non-interpretable, and FF was not reported. However, this was considered an indication that a RAA was present, and another bioinformatic software was then utilized to establish which chromosome was involved. These non-interpretable FF cases were denoted as “FF unavailable” in the Results section of the manuscript.

### Clinical outcomes collection

Follow-up was attempted for all cases and was carried out according to the individual procedures of each laboratory or clinic. Clinical outcome information included diagnostic procedures, namely chorionic villus sampling (CVS) and amniocentesis, products of conception (POC) testing, findings from ultrasound examinations, newborn physical exam information, and/or any testing performed on the newborn. Desired clinical outcome data included baseline demographic details, adverse pregnancy outcomes, birth weight, or outcomes from the ongoing pregnancy (such as serial growth and any newborn complications). Cases were considered to have had confirmatory fetal testing if amniocentesis, POC testing, or newborn testing (blood test or umbilical cord test) had been carried out. Cases were considered to have undergone confirmatory placental testing if CVS or placental testing at birth had been carried out. Cases were deemed to be concordant if they involved a full or mosaic RAA or UPD on the chromosome of interest.

## Results

### Patient and sample details

In our study, a total of five sites provided details on 109 patients that received a high-risk screening result for a rare autosomal aneuploidy following genome-wide cfDNA screening, including 20 cases from Site A, 6 from Site B, 66 from Site C, 12 from Site D, and 5 cases from Site E. The five study sites were from multiple geographical regions, namely Australia, Canada, Argentina, and South Africa. Patients that underwent cfDNA screening from 2015 to 2021 were included, with 2020 having the highest percentage of cases included in the study cohort (23.9%; Supplementary Figure S1). Patient demographics are shown in Table 1; mean maternal age was 36.1 years, and mean gestational age was 11.9 weeks. The vast majority of patients (107, 98.2%) had a singleton pregnancy; both ongoing twin cases were dichorionic twin pregnancies. As can be seen from Table 1, maternal age was the most common referral indication (58.7%) followed by patient preference (32.1%). For a number of cases, we reassigned the referral indication based on additional information as detailed in Table 1 footnotes. Information on whether conventional screening was performed was available for 95 (87.2%) patients in the study cohort; of these, 19 (17.4%) had conventional screening and 76 (69.7%) did not. Of the 76 cases where conventional screening was not carried out, PAPP-A results were provided for 17 cases and NT results were provided for 20 cases. Overall, NT results were available for 29 (26.6%) patients in our cohort, with 27 (93.1%) of these cases having a normal NT result (<3.5 mm).

**TABLE 1:** Demographics of the study cohort (*n* = 109).

Variable	Value
Maternal Age, yr	
Mean	36.1
Median	37
Range	25–47
Gestational Age, wk	
Mean	11.9
Median	11.1
Range	10–22.1
Basis of Gestational Age, *n* (%)	
Based on LMP	9 (8.3%)
Based on USS	100 (91.7%)
Type of Pregnancy, *n* (%)	
Singleton^a^	107 (98.2%)
Twins	2 (1.8%)
Referral Indications, *n* (%)	
Abnormal Ultrasound	3 (2.8)
Maternal Age^b^	64 (58.7)
Family History^c^	2 (1.8)
Patient Preference^d^	35 (32.1)
Multiple Indications^e^	5 (4.6)

LMP, last menstrual period; USS, ultrasound scan.

a Includes four cases of demised twin which occurred prior to the cfDNA screening blood draw.

b Thirty-eight of these cases had no known indication on the test requisition form (TRF). As the maternal age was over 35 years, we reassigned them as Maternal Age.

c For one case, family history details listed sensory motor neuropathy with or without agenesis of the corpus callosum. For the second case, there was a previous affected child/pregnancy (the child is being investigated for Prader-Willi syndrome and other genetic syndromes).

d Twenty-four of these cases had no indication listed on the TRF. As the maternal age was less than 35 years, we reassigned them as Patient Preference cases. For the remaining 16 cases, the TRF listed the referral indication as Other, with primary screening test as the detail provided. We reassigned these cases as Patient Preference cases as well.

e Two of these cases were originally entered as advanced maternal age on the TRF. As one of the cases also had an NT of 4.6 mm following ultrasound, and the other case had a previous affected child/pregnancy we reassigned both cases as Multiple Indications. The other three cases were listed as Other on the TFF. As one patient had a maternal age over 35 years of age and had a previous failed cfDNA screen at a different provider, we reassigned this case as a Multiple Indications case. The other two patients had a positive conventional screening result and advanced maternal age.

### Genome-wide cfDNA screening results

**FIGURE 1:**
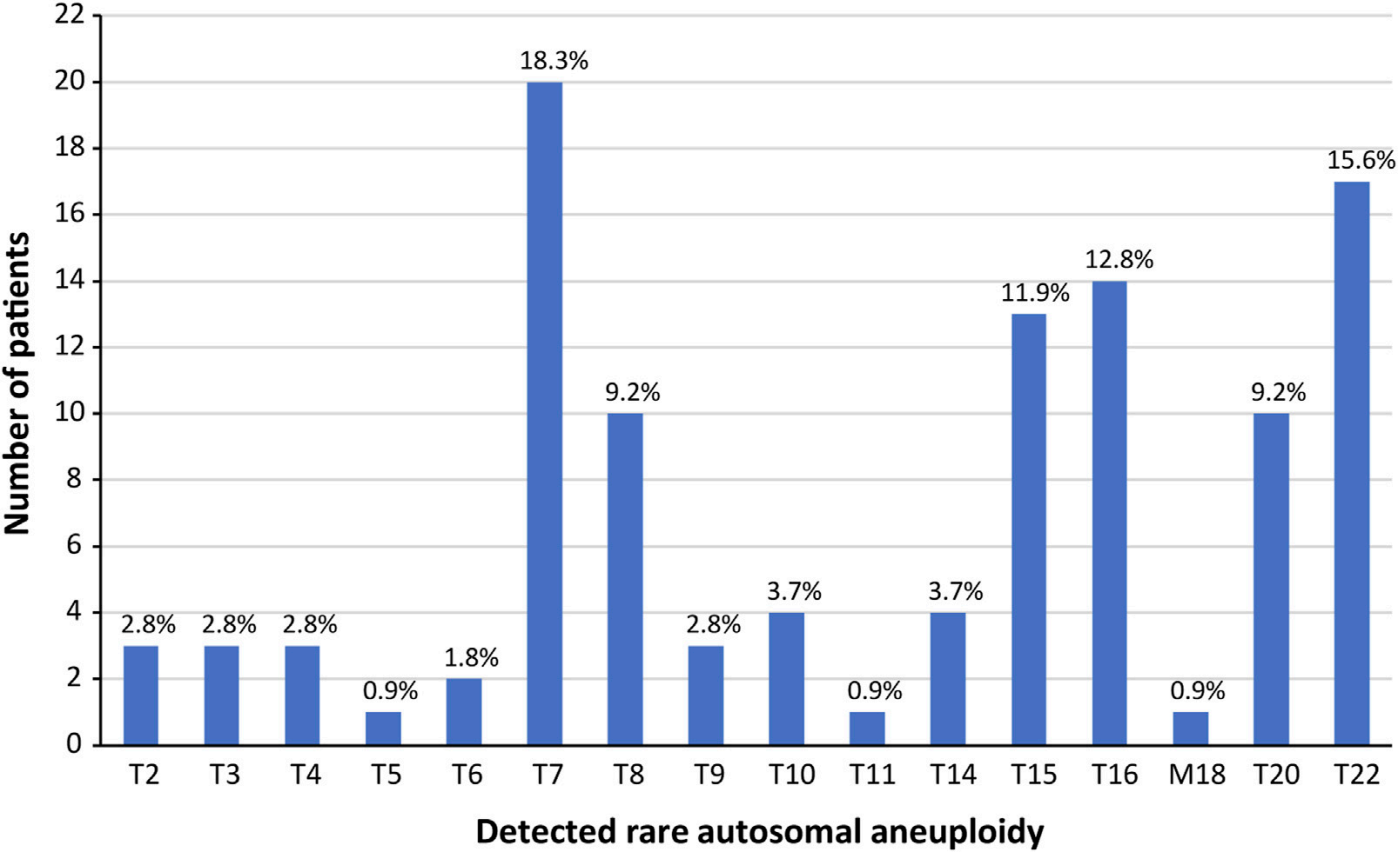
Detected rare autosomal aneuploidies in the study cohort. There were no rare autosomal aneuploidies observed on chromosomes 1, 12, 17, or 19; trisomies observed on chromosomes 13, 18, or 21 were not included.

Figure 1 shows the distribution of RAAs (i.e., aneuploidies that were not trisomy 21, 18, or 13) across chromosomes in our patient population; no RAAs were found for chromosomes 1, 12, 17, and 19. There was only one monosomy case in our cohort (monosomy 18); the rest were all trisomy cases. Trisomy 7 was the most common RAA (*n* = 20; 18.3%), followed by trisomy 22 (*n* = 17; 15.6%) and trisomy 16 (*n* = 14; 12.8%). For the twin pregnancies, a trisomy 15 and a trisomy 22 were reported. There were four cases that were listed as singleton pregnancies that had a demised twin prior to the first cfDNA screening blood draw. All four of these cases (2x T15, 1x T22, and 1x T16) had a repeat blood draw with resolution of the aneuploidy, and normal pregnancy/birth outcomes. Diagnostic testing was only performed in one case and was normal for the ongoing twin. For additional details on these four cases, please *see* Appendix A (Data Sheet 1) in the Supplementary Materials. It is therefore reasonable to assume that in all four cases, the initial RAA noted on cfDNA screening may be attributed to the demised twin.

**FIGURE 2:**
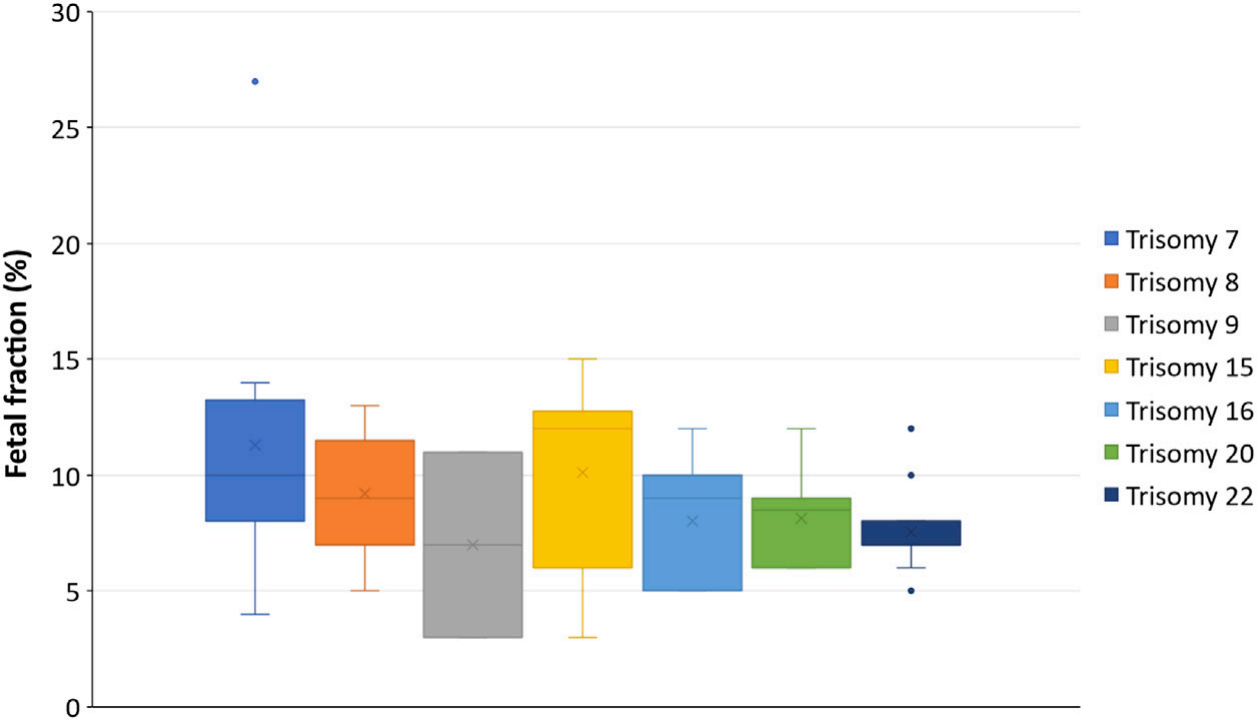
Relationship between fetal fraction and RAAs per chromosome. The box plots represent the first and third quartile (upper and lower margins of the box, respectively), the minimum and maximum FF values (lower and upper whiskers, respectively), the median FF (horizontal line within the box), and the mean FF (X within the box). The dots are outliers.

The fetal fraction (FF) was available for 67 (61.5%) cases in our study cohort and ranged from 3% to 27%, with both an average and a median of 9%. The fetal fraction distribution for these 67 cases across each of the affected chromosomes is shown in Supplementary Figure S2. We have categorized these 67 cases into cases that were concordant with the confirmatory fetal testing, concordant with the confirmatory placental testing, and the unconfirmed cases (includes cases where the RAA was not detected in the fetus but the placenta was not tested). We also highlighted the two discordant cases that underwent both confirmatory fetal and placental testing; further information on diagnostic outcomes is provided in the following section. There were seven chromosomes that had at least three RAA cases with FF information (Figure 2). Although the range of FF varied between these chromosomes, all cases reported a FF between 3% and 15% (except for the outlier of 27% for one of the trisomy 7 cases). It should be noted that all cases included in Figure 2 were singleton cases that underwent cfDNA screening in the first trimester. When we looked at these seven affected chromosomes only (chromosomes 7, 8, 9, 15, 16, 20, and 22), we found that there were also five RAA cases with known FFs from patients with singleton pregnancies that had undergone cfDNA screening in the second trimester (not shown in Figure 2). These included two trisomy 7 cases (13% FF; 15% FF), one case of trisomy 8 (9% FF), one case of trisomy 16 (14% FF), and one case of trisomy 22 (7%).

### Diagnostic outcome data

**FIGURE 3:**
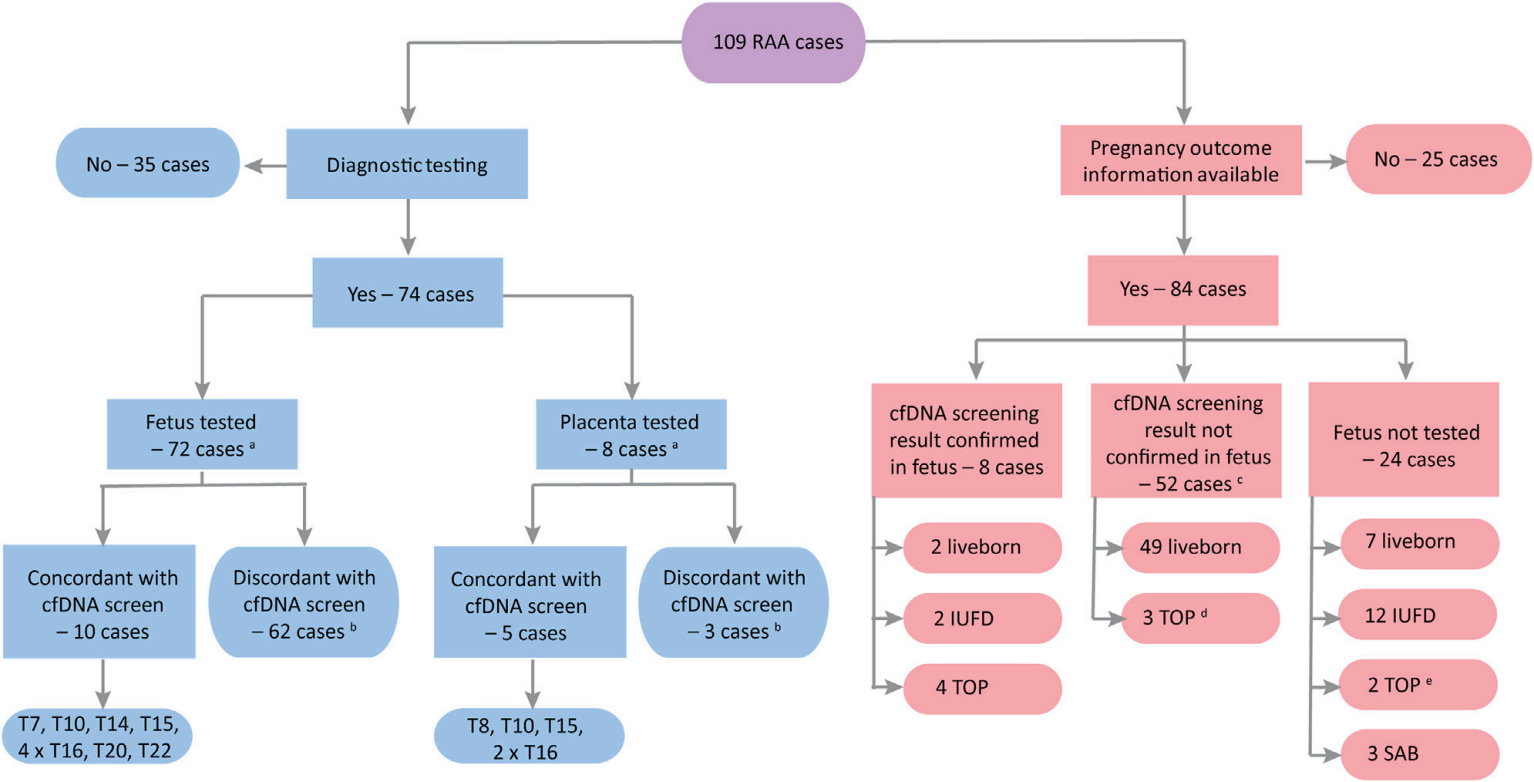
Flowchart of outcomes for the study cohort (*n* = 109). IUFD, intrauterine fetal demise; PX, pregnancy; SAB, spontaneous abortion; TOP, termination of pregnancy. ^a^Six cases underwent both fetal and placental testing. ^b^In two cases the cfDNA screening result was discordant with both fetus and placenta. ^c^The cfDNA screening result was confirmed in placental tissue in four of these cases: three liveborn (trisomy 8 case with FGR and premature birth; trisomy 16 case with spontaneous premature birth; trisomy 16 case with induced premature birth for preeclampsia) and one selective TOP (trisomy 15 with severe FGR). ^d^The other two TOP cases were a trisomy 20 case with multiple abnormalities noted on autopsy and a trisomy 7 case with cleft lip/palate identified on ultrasound following the cfDNA screen. ^e^One case (trisomy 7) had a mutation in SLC12A6; the other case (trisomy 8) had abnormalities but no further details are available.

Figure 3 shows a flowchart of the outcomes for all 109 RAA cases, with details in Supplementary Table S1. Overall, 74/109 patients (67.9%) underwent some type of diagnostic testing, with most undergoing amniocentesis (Supplementary Table S2). The RAA was confirmed in 10/72 (13.9%) patients where fetal testing was performed. Six patients had both fetal and placental testing, and an additional two patients had placental testing only. The RAA was confirmed in five of these eight patients (62.5%), indicating CPM as the underlying etiology for positive NIPT. Thus, based on these findings, fifteen cases could be considered to be concordant (either fetal or placental) with the diagnostic testing; the diagnostic yield was therefore 20.3% (15/74; *see* Table 2 for details of these cases). There were no cases in our cohort where the RAA was found to be present in both the fetus and the placenta following diagnostic testing.

**TABLE 2:** Details of concordant cases.

Observed concordance	Case no.	cfDNA screening result	Fetal fraction (%)	Interventions prompted	Pregnancy outcome	Pregnancy complications	Newborn physical exam
Fetus (mosaic)	1	Trisomy 7	13	TOP	Elective TOP	—	—
Fetus (mosaic)	2	Trisomy 10	N/a	TOP	Elective TOP	—	—
Fetus	3	Trisomy 14	N/a	Fetal anatomy scan	IUFD	—	—
Fetus (mosaic/UPD)	4	Trisomy 15	N/a	TOP	Elective TOP	—	—
Fetus (mosaic)	5	Trisomy 16	5	TOP	Elective TOP	—	—
Fetus (UPD)	6	Trisomy 16	9	Alteration of pregnancy monitoring^a^	Liveborn^b^	Preeclampsia	FGR, prematurity, cleft palate
Fetus (mosaic)	7	Trisomy 16	N/a	Alteration of pregnancy monitoring^a^	Unknown (lost to follow-up)	Unknown	Unknown
Fetus (mosaic)	8	Trisomy 16	9	Alteration of pregnancy monitoring^a^	Unknown (lost to follow-up)	Unknown	Unknown
Fetus (mosaic)	9	Trisomy 20	N/a	Alteration of pregnancy monitoring^a^	Liveborn^c^	None	Normal. Baby required breathing support at birth but was otherwise well
Fetus	10	Trisomy 22	N/a	Other	IUFD	—	—
Placenta (mosaic)	11	Trisomy 8	N/a	Alteration of pregnancy monitoring^a^	Liveborn^d^	Severe FGR	Normal
Placenta (mosaic)	12	Trisomy 10	N/a	Alteration of pregnancy monitoring^a^	Unknown (lost to follow-up)	Unknown	Unknown
Placenta	13	Trisomy 15	N/a	Alteration of pregnancy monitoring^a^	Elective TOP	Severe FGR	Hypospadias
Placenta (mosaic)	14	Trisomy 16	N/a	Alteration of pregnancy monitoring^a^	Liveborn^e^	Preeclampsia	No information provided
Placenta (mosaic)	15	Trisomy 16	10	—	Liveborn^f^	None	No information provided

FGR, fetal growth restriction; IUFD, intrauterine fetal demise; N/a, not available; TOP, termination of pregnancy.

a Monitoring for fetal growth and adverse pregnancy outcomes.

b Induced premature birth (27–32 weeks).

c Delivery at term (38–42 weeks).

d Induced premature birth at 36 weeks due to FGR.

e Induced premature birth at 34 weeks due to preeclampsia.

f Spontaneous premature birth (33–37 weeks).

In addition, UPD testing was carried out for 18 cases in our study cohort, including eight trisomy 7 cases, one trisomy 14 case, six trisomy 15 cases, two trisomy 16 cases, and one trisomy 20 case. There was one case of maternal UPD 15 and one case of maternal UPD 16 reported; these two cases are included in the 15 confirmed concordant cases.

**FIGURE 4:**
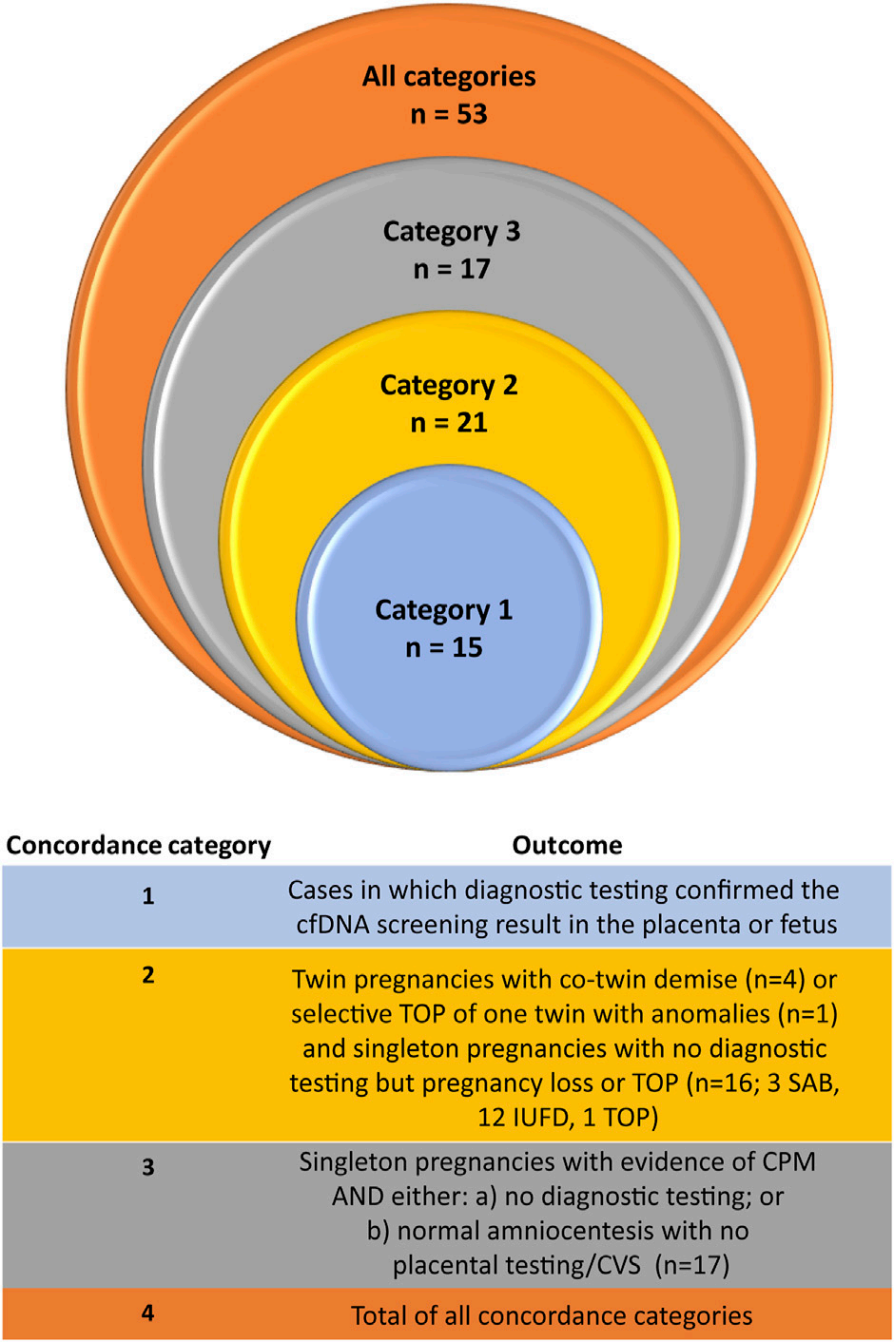
Estimation of concordance in the study cohort.

Moreover, there are a number of other cases in the total cohort (109 cases) where it is possible that a RAA was present in either the fetus or the placenta (*see* Figure 4). This includes the four demised twin cases [*see* Appendix A (Data Sheet 1) of the Supplementary Materials], where the initial cfDNA screen reported a RAA but follow-up cfDNA screening was normal. In one of the dichorionic twin cases, which reported a trisomy 22 by cfDNA screening, there was a selective termination of one of the twins following cfDNA screening but prior to diagnostic testing. That twin had cystic hygroma on ultrasound at 13 weeks as well as delayed growth and possible brain anomalies. Amniocentesis was carried out on the surviving twin only, which was found to be normal and there was a normal birth outcome. Amongst the singleton cases with no diagnostic testing, there were three cases of a spontaneous miscarriage (one trisomy 9 and two trisomy 22), twelve cases with an IUFD (one trisomy 7, three trisomy 15, two trisomy 16, one trisomy 20, and five trisomy 22 cases), and one case of an elective termination of pregnancy (TOP) due to fetal anomalies. If we include these additional 21 cases (Figure 4, category 2), then the concordance would increase to 33.0% (36/109). There were also 17 singleton cases that underwent diagnostic fetal testing (RAA not detected in the fetus), but not placental testing, that experienced adverse pregnancy outcomes (such as FGR, preeclampsia, or preterm birth) or that ended with a termination of the pregnancy. Both of the elective TOP cases had anomalies noted on ultrasound (Figure 4, category 3). If we include these additional 17 cases, then the concordance could be as high as 53/109 (48.6%).

### Pregnancy outcomes

**FIGURE 5:**
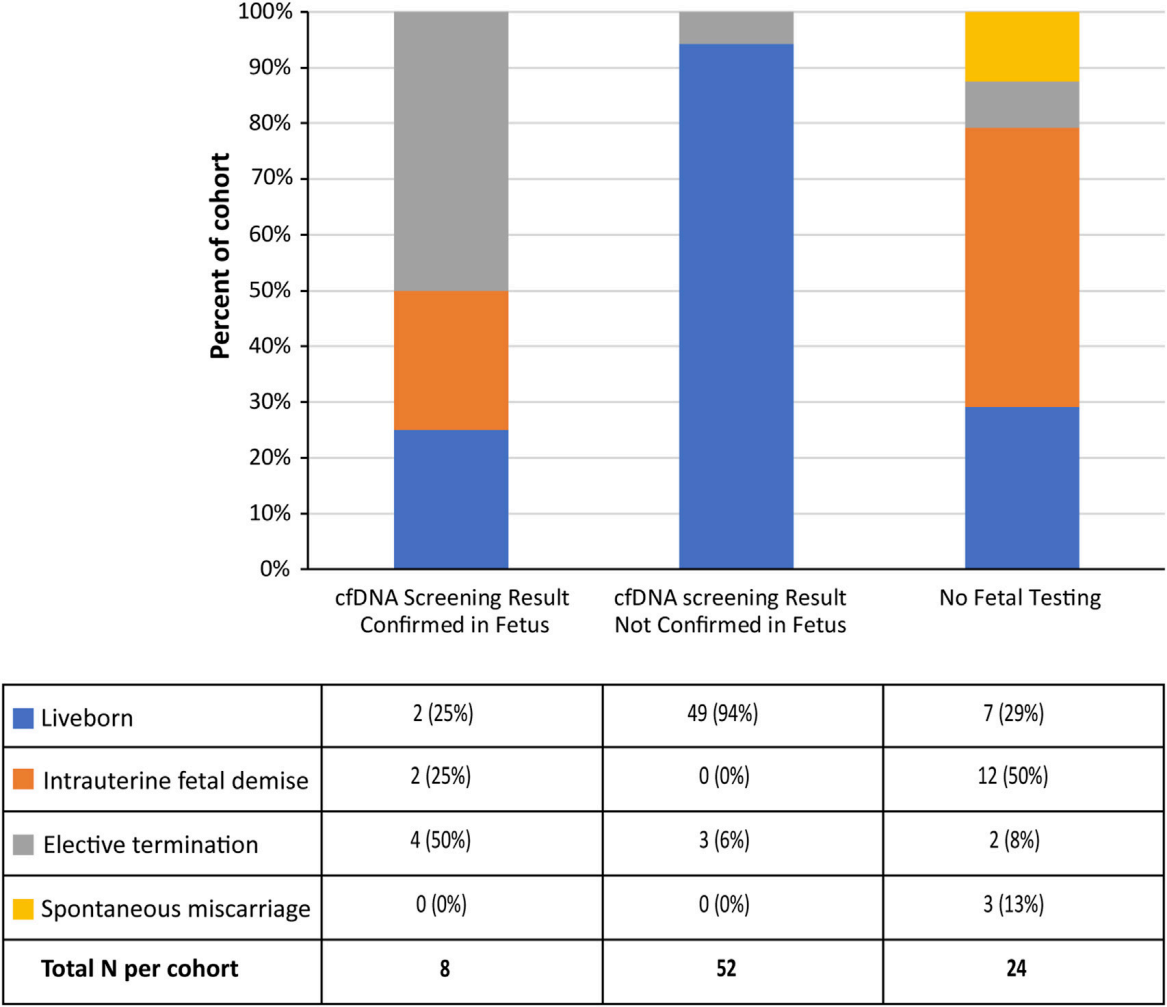
Pregnancy outcomes for the study cohort (*n* = 84).

Pregnancy outcome data was available for 84 cases in our cohort (Supplementary Table S1 and Figure 5). This included eight of the ten fetal concordant cases; further details of these cases are provided in Table 2. There were also 52 cases with pregnancy outcomes where the cfDNA screening result was not confirmed in the fetus. Of these 52 cases, three patients had preeclampsia, ten experienced FGR, and in one other case the placenta was reported as being “grossly abnormal.” Although the majority of these cases delivered at term, there were a number of cases that underwent an induced preterm delivery (Supplementary Table S1), and three of these patients had an elective termination of pregnancy. All three cases that underwent an elective TOP had anomalies noted on ultrasound or autopsy. The vast majority of these cases (46/52; 88.5%) had not undergone confirmatory placental testing.

In addition, there were 24 cases with pregnancy outcomes where fetal confirmatory testing had not been carried out. Of these 24 cases, 12 (50%) experienced IUFD (one trisomy 7, three trisomy 15, two trisomy 16, one trisomy 20, and five trisomy 22 cases), three cases (13%) experienced a spontaneous miscarriage (one trisomy 9 and two trisomy 22 cases), and two cases had an elective termination of pregnancy (one case had anomalies noted on ultrasound and the other case had a mutation in the SLC12A6 gene on prenatal diagnosis). The remaining seven cases were liveborn; of these seven liveborn cases, three were co-twin demise cases (discussed previously), one was an induced premature case with FGR, and another was a spontaneous premature case. Therefore, only two of the cases in this subcohort had no confirmed pregnancy complications.

### Relationship between RAAs, low PAPP-A, and pregnancy outcomes

PAPP-A results were available for 29 patients in our cohort, of which 10 (34.5%) had low (<0.5 MoM) or very low (<0.2 MoM) PAPP-A values (Table 3). Two of these cases had a confirmed RAA (trisomy 16) in the fetus, with both reporting very low PAPP-A values (0.04 and 0.12). None of the 10 cases had placental testing for presence of aneuploidy. Fetal growth restriction was observed in four of these patients (one trisomy 2, one trisomy 7, one trisomy 15, and one trisomy 16). Overall, six of the ten cases noted pregnancy complications and one other case was an elective TOP (confirmed trisomy 16) which may have had complications that were not noted for the study. Of the 19 cases with normal PAPP-A values, fifteen underwent diagnostic testing—fourteen had amniocentesis with one case of confirmed RAA in the fetus (mosaic trisomy 16 [20%–30%]). The other case had postnatal testing of newborn blood (no aneuploidy detected) and placental tissue (which confirmed mosaic Trisomy 16 in the placenta). Two of these 19 cases experienced IUFD, one had a low-lying placenta, two had preeclampsia, and one had presence of fibroma.

**TABLE 3:** Relationship between RAAs, low PAPP-A, and Pregnancy Outcomes.

PAPP-A	cfDNA screening result	Confirmed in fetus^a^	Pregnancy complications	Pregnancy outcomes
0.04	Trisomy 16	Yes	Preeclampsia	Induced (premature at 27–32 weeks);
				FGR
0.12	Trisomy 16	Yes	None reported	Elective TOP
0.14	Trisomy 7	No	FGR	Liveborn (spontaneous premature 33–37 weeks)
0.15	Trisomy 8	No	None reported	Liveborn (term)
0.15	Trisomy 2	No	FGR	Liveborn (spontaneous premature 33–37 weeks)
0.24	Trisomy 9	No^b^	Gestational diabetes	Liveborn (term)
0.25	Trisomy 16	No^c^	None reported	Liveborn (term);
				child has developmental delay
0.39	Trisomy 3	No	None reported	Liveborn (term)
0.4	Trisomy 15	No	FGR	Liveborn (term)
0.48	Trisomy 7	No	Irritable uterus, multiple admissions for preterm labor, induced for reduced fetal movement	Liveborn (term)

FGR, fetal growth restriction; NBS, newborn screening; PAPP-A, pregnancy associated plasma protein-A; TOP, termination of pregnancy.

a None of the cases underwent placental testing.

b 2p22.1 duplication in fetus (305 kb).

c Deletion in chromosome region 16p13.11 in the fetus.

## Discussion

Here, we discuss a multi-site global study looking at the clinical implications of prenatal screening for RAAs by genome-wide cfDNA screening. Our study showed the wide array of situations and outcomes that can occur when a patient receives a high-risk call for a RAA following cfDNA screening, reflecting the real-world experiences of prenatal screening. We found that early identification of these aneuploidies by genome-wide cfDNA screening was beneficial in a variety of clinical situations. It allowed for a change in pregnancy management in a number of cases (e.g., alteration of pregnancy monitoring for fetal growth and adverse pregnancy outcomes) and also provided a possible explanation for cases of miscarriage, co-twin demise, and fetal death as well as other pregnancy complications.

Trisomies 7, 22, and 16 were the most commonly observed trisomies in our study cohort. Trisomy 7 was also found to be the most commonly observed RAA in several other studies (Pertile et al., 2017; Scott et al., 2018; van der Meij et al., 2019; Van Den Bogaert et al., 2021). Trisomy 16 was the most common trisomy to be confirmed in either the fetus or the placenta (40% of all cases) in our cohort, with one case confirmed as UPD and the rest confirmed as mosaic trisomy 16. This is not surprising given that trisomy 16 is believed to be the most common trisomy, occurring in at least 1% of clinically recognized pregnancies (Hassold et al., 1995). Trisomy 16 is associated with a high probability of fetal death, fetal growth restriction, fetal anomalies, and preterm delivery (Benn, 1998; Peng et al., 2021). A study by Yong *et al.* (Yong et al., 2003) found that the level of trisomy in the different fetal or placental tissues was an indicator of the severity of outcomes. Unfortunately, we did not have complete outcomes for a number of our confirmed trisomy 16 cases.

In our study, ten of the 72 cases (14%) that underwent fetal diagnostic testing had a confirmed fetal RAA, with the majority of these cases ending in either an elective termination of pregnancy or fetal demise, highlighting the importance of this knowledge to the patient. As noted above, the true concordance could not be determined due to the fact that approximately a third of patients did not undergo confirmatory fetal testing and over 90% of patients did not have confirmatory placental testing. However, based on adverse pregnancy outcomes observed in many of the patients, there were many other cases where the RAA may have been present in either the fetus or the placenta. When we included the placental concordant cases, our overall rate of concordance was 20.3%. A recent study by Soster et al. (Soster et al., 2021) noted a positive predictive value of 22.4% for rare autosomal trisomies in a large cohort of genome-wide cfDNA screening samples. Although the PPV for rare autosomal aneuploidies is not as high as that for common trisomies, it is still approximately four-fold higher than the PPV observed (3.4%) using conventional screening for trisomies 21 and 18 (Gregg et al., 2016). Of the five confirmed CPM cases in our cohort, two had severe FGR and one had a spontaneous premature birth (outcomes for the remaining two cases are unknown), illustrating the impact that a placental RAA can have on pregnancy outcomes. A recent literature review by Eggenhuizen *et al.* (Eggenhuizen et al., 2021) looking at the association between CPM and adverse pregnancy outcomes found that CPM was associated with fetal growth restriction, preterm birth, structural fetal anomalies, and pregnancy complications such as preeclampsia. A number of these adverse outcomes were also noted in many of our study patients.

Our study clearly illustrates there can be value in genome-wide cfDNA screening for many patients, particularly in cases of pregnancy loss. Even in cases where diagnostic testing is not carried out, having a high-risk assessment for a RAA based on cfDNA screening may provide some explanation in the event of a pregnancy loss, which could be of value to patients. In our study cohort, we reported four cases of a co-twin demise, three cases of spontaneous miscarriage, and fourteen cases of IUFD. As noted above, in all four of the co-twin demise cases a follow-up cfDNA screen at a later gestational date did not show presence of a RAA, providing a likely explanation to the patient for loss of that twin. None of the spontaneous miscarriage cases underwent diagnostic testing. Although none of the IUFD cases had diagnostic testing prior to loss of the fetus, two of these 14 cases had follow-up POC analysis which showed presence of the RAA (trisomy 14 and trisomy 22, respectively) in the fetus. Genome-wide cfDNA screening can also be beneficial in altering the pregnancy management of the patient with the potential to offer increased and earlier ultrasounds and diagnostic procedures. In addition, a high-risk call for a RAA on a cfDNA screen can provide a possible explanation for pregnancy complications such as fetal growth restriction and preeclampsia.

Detailing outcomes for RAA cases can be very challenging in a study such as this one. This is due to the varying number of RAA cases per affected chromosome, the different approaches taken for pregnancy management of these patients, the differences in the outcome data collected and available at each of the contributing sites, and the low prevalence of RAAs in general. This has also been reported in single site studies of RAAs identified by cfDNA screening, with studies frequently reporting incomplete outcomes and varied clinical management practices (Liang et al., 2018; Kleinfinger et al., 2020; Soster et al., 2021). Whilst there is a myriad of ways that these outcomes could be grouped and analyzed, we chose to group patients into three different tiers as noted in Figure 4. The first tier (category 1) in our system represents patients where diagnostic testing found presence of the RAA in the fetus and/or placenta. The second tier (category 2) denotes cases where the high-risk RAA cfDNA screening result provided potentially valuable information for the patient, with outcomes that include pregnancy loss and elective termination of pregnancy following the identification of ultrasound anomalies. The final third tier (category 3) represents cases where the cfDNA screening result could be beneficial for the healthcare professional, as it indicated the need to monitor the pregnancy closely for complications such as fetal growth restriction and preeclampsia. Although our approach is subjective, this tiered system could be a useful framework for healthcare professionals in determining the clinical utility of screening for RAAs.

One of the strengths of our study is that it is a global multi-centre study where a broad spectrum of care was received by patients undergoing cfDNA screening. This includes the use of different patient protocols and techniques for follow-up testing and analysis, as well as varying clinical practices for pregnancy management in these patients. We also had a large number of patients with clinical outcome information, including diagnostic outcomes and/or pregnancy outcomes. A limitation of our study is that we only had a small number of rare autosomal aneuploidies on some chromosomes and no aneuploidies observed on other chromosomes, preventing us from drawing conclusions regarding the clinical impact of RAAs on individual chromosomes. Due to the low prevalence of RAAs, this will be a limitation of all studies looking at RAAs. As our study was a retrospective analysis and full outcomes were not available for all of the cases, we were also not able to make an accurate determination of the true rate of pregnancy complications and adverse pregnancy outcomes in our study population. Both of these limitations could be addressed in future studies that focus on either a particular rare autosomal aneuploidy or a particular type of outcome observed in these patients such as FGR or IUFD. In addition, there may have been ascertainment bias regarding which cases had placental testing, which may have also been influenced by the clinical protocols in place at each site. Due to the small number of cases that did undergo placental testing, it was not possible to make any statistical comparisons between known CPM cases and suspected CPM cases, or cases where CPM was ruled out. Finally, a large number of patients were of a slightly higher maternal age than would be observed in a general pregnancy cohort, with over 60% of study participants listing maternal age as the referral indication for genome-wide cfDNA screening.

A large number of studies have been published in recent years looking at the identification of RAAs by genome-wide cfDNA screening. There is a lot of variation in these studies, including type of patient population, number of samples tested, and availability of outcome information (such as diagnostic testing for presence of RAAs, UPD testing, and adverse pregnancy outcomes). We attempted to capture the large amount of data that currently exists in the literature by compiling a table (Supplementary Table S3) that provides an overview of many of these recent studies. We carried out this multi-site global study to not only add to this growing body of evidence, but also to provide more information on the pregnancy and birth outcomes experienced by patients with a high-risk call for a RAA following genome-wide cfDNA screening, as these outcomes are not provided in many of the other studies to date.

**FIGURE 6:**
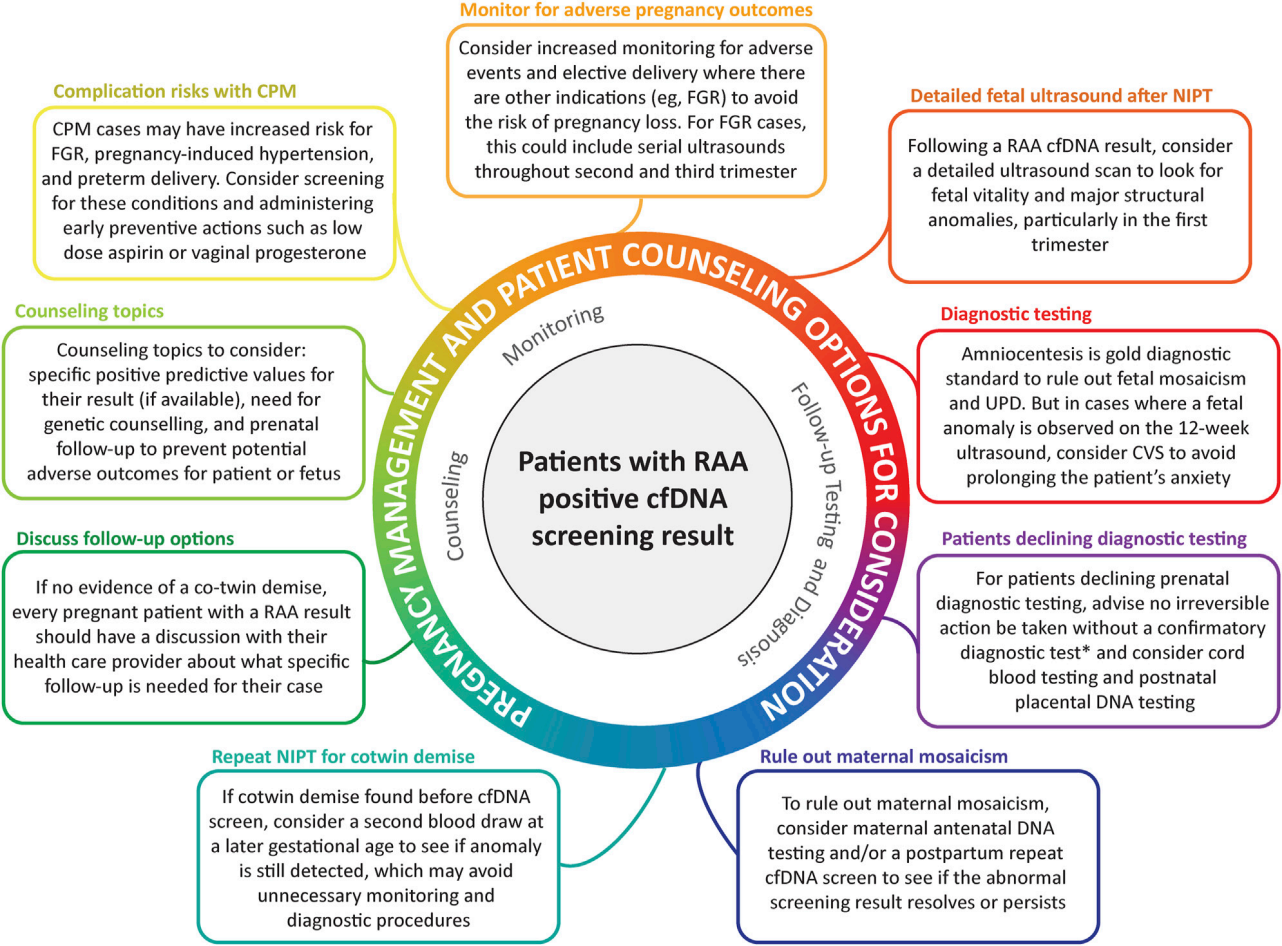
Pregnancy management and patient counselling options for consideration. *NIPT is a screening test. No irreversible clinical decisions should be made based on these screening results alone.

Based on the results of our study, information from previous publications identified through the literature review that we carried out, and the experiences of members of our Consortium, some options for pregnancy management and patient counseling have been considered, with recognition that further research in this area is required to confidently establish an appropriate approach. These considerations are outlined in Figure 6.

In conclusion, our study has shown that genome-wide screening for presence of rare autosomal aneuploidies can be beneficial in a number of clinical situations, such as providing a possible explanation for an adverse pregnancy outcome or resulting in a change in pregnancy management. These interventions and possible explanations for pregnancy outcomes are of great benefit to pregnant patients, allowing for increased monitoring throughout the pregnancy or potentially alleviating any feelings of perceived personal responsibility for adverse outcomes. It can also be valuable for future pregnancies to determine if there is a recurrence risk for the anomaly in question. The recurrence risk may be low in many cases, which in itself can be valuable information in terms of the patient’s anxiety and future pregnancy planning. This multi-site global study adds to the growing body of evidence regarding genome-wide cfDNA screening, and also adds valuable information regarding the clinical outcomes of patients that receive a high-risk screening call for a rare autosomal aneuploidy.

## Data Availability

The data that support the findings of this study are available upon reasonable request from the corresponding author. The clinical outcome data in this study was obtained from patient records and therefore will not be made available through a database because of privacy concerns as well as ethical restrictions. However, deidentified data that underlie the results reported in this article (text, figures, tables, and appendices) are available by reasonable request within 12 months of publication. Proposals must include a detailed and sound methodological approach, including a statistical analysis plan, as would be reasonably required for the purposes of publication in a peer-reviewed scientific journal.
