# Supplementary material for: Multisite assessment of the impact of cell-free DNA-based screening for rare autosomal aneuploidies on pregnancy management and outcomes
Source: Front Genet. 2022 Aug 29;13:975987. doi: 10.3389/fgene.2022.975987 (PMC9465083; doi:10.3389/fgene.2022.975987)
Supplement: Supplementary file 1 [file Table1.DOCX]

Supplementary Material

## Supplementary Table 1. Diagnostic and pregnancy outcomes for the study cohort (n=109).

| **RAA (n)** | ***With outcome* \| lost to f/u** | **Diagnostic testing** | **Fetal testing** | **Placental testing** | **Confirmed in fetus** | **Confirmed in placenta** | **Diagnostic testing details** | **With obstetric outcome** | **Normal Livebirth/Neonate** | **Pregnancy complicat-ions** | **PE** | **Preterm birth** | **Abnormal newborn exam** | **Pregnancy loss or stillbirth** | **FGR** | **SA** | **TAB** |
| --- | --- | --- | --- | --- | --- | --- | --- | --- | --- | --- | --- | --- | --- | --- | --- | --- | --- |
| T2 (3) | ***2*** \| 1 | 2 | 2/2 | 0/2 | 0/2 | ----- | AF-Array (1)  AF-FISH and array (1) | 2 | 1 | 1^A^ | 0 | 1^A^ | 0 | 0 | 1^A^ | 0 | 0 |
| T3 (3) | ***3*** \| 0 | 3 | 3/3 | 0/3 | 0/3 | ----- | AF-Array (2)  AF-qfPCR, Karyo, aCGH (1) | 2 | 2 | 0 | 0 | 0 | 0 | 0 | 0 | 0 | 0 |
| T4 (3) | ***3*** \| 0 | 3 | 3/3 | 0/3 | 0/3 | ----- | AF-Array (3) | 3 | 1 | 2^B,C^ | 0 | 2^B,C^ | 0 | 0 | 1^C^ | 0 | 0 |
| T5 (1) | ***1*** \| 0 | 1 | 1/1 | 0/1 | 0/1 | ----- | AF-Karyo (1) | 1 | 0 | 1^D^ | 0 | 1^D^ | 0 | 0 | 1^D^ | 1^D^ | 0 |
| T6 (2) | ***1*** \| 1 | 0 | ----- | ----- | ----- | ----- | ----- | 1 | 1 | Unknown^E^ | 0 | 0 | 0 | 0 | 0 | 0 | 0 |
| T7 (20) | ***19*** \| 1 | 16 | 15/16 | 1/16 | 1/15 | 0/1 | CVS-qfPCR + Karyo (1)  AF-qfPCR (1)  AF-Karyo (1)  AF-Array (5)  AF - Array + UPD (4)  AF - FISH + UPD (1)  AF - FISH, Karyo, aCGH, UPD (1)  NBB-Array + UPD (1)  UC + CB-Array + UPD (1) | 18 | 8^F^ | 10^G,H,I,J,K,L,M,N,O,P^ | 0 | 4^I,J,K,L^ | 0 | 1^M^ | 2^I,K^ | 1^N^ | 3^N,O,P^ |
| T8 (10) | ***10*** \| 0 | 9 | 9/9 | 1/9 | 0/9 | 1/1 | AF-Karyo (1)  AF-Array (6)  NBB-Array (1)  UC-FISH + Array + Postnatal placenta - FISH (1) | 9 | 7^Q^ | 2^R,S^ | 0 | 1^R^ | 0 | 0 | 1^R^ | 1^S^ | 1^S^ |
| T9 (3) | ***2*** \| 1 | 1 | 1/1 | 0/1 | 0/1 | ----- | AF-Array (1) | 2 | 1 | 1^T^ | 0 | 0 | 0 | 1^T^ | 0 | 0 | 0 |
| T10 (4) | ***4*** \| 0 | 4 | 3/4 | 1/4 | 1/3 | 1/1 | AF-Karyo (1)  AF-Array (1)  AF-FISH, qfPCR, Array (1)  CVS-Array (1) | 1 | 0 | ----- | ----- | ----- | ----- | ----- | ----- | ----- | 1^U^ |
| T11 (1) | ***0*** \| 1 | 0 | ----- | ----- | ----- | ----- | ----- | 0 | ----- | ----- | ----- | ----- | ----- | ----- | ----- | ----- | ----- |
| T14 (4) | ***4*** \| 0 | 4 | 4/4 | 0/4 | 1/4 | ----- | AF-Karyo (2)  AF-Karyo + UPD (1)  POC-Karyo (1) | 3 | 2 | 1^V^ | 0 | 0 | 0 | 1^V^ | 0 | 0 | 0 |
| T15 (13) | ***12*** \| 1 | 8 | 8/8 | 1/8 | 1/8 | 1/1 | AF-Karyo (3)  AF-Array (1)  AF-FISH + UPD (1)  AF-Array + UPD (2)  AF -FISH + Array + UPD + POC-CV + UC-Array (1) | 10 | 4^W^ | 6^X,Y,Z,AA^ | 0 | 0 | 1^X^ | 3^Y^ | 2^X,Z^ | 0 | 2^X, AA^ |
| T16 (14) | ***12*** \| 2 | 8 | 8/8 | 2/8 | 4/8 | 2/2 | AF-Array (3)  AF-Array + UPD (1)  AF-FISH +Array (1)  Postnatal placenta + UC + CV -NGS (1)  NBB-Array + UPD (1)  NBB-karyo + FISH + postnatal placenta-array (1) | 10 | 2^BB,CC^ | 8^DD,EE,FF,GG,HH,II,JJ^ | 3^DD,EE,FF^ | 5^DD,EE,FF,GG,HH^ | 0 | 2^II^ | 1^HH^ | 0 | 1^JJ^ |
| M18 (1) | ***1*** \| 0 | 1 | 1/1 | 1/1 | 0/1 | 0/1 | AF-Karyo + postnatal placenta (1) | 1 | 1 | 0 | 0 | 0 | 0 | 0 | 0 | 0 | 0 |
| T20 (10) | ***10*** \| 0 | 9 | 9/9 | 1/9 | 1/9 | 0/1 | AF-Karyo (3)  AF-Array (2)  AF-Array + FISH + POC umbilical cord (1)  AF-Array + UPD (1)  AF-Unspecified (1)  AF-Karyo + Postnatal placenta-Karyo (1) | 9 | 4 | 5^KK,LL,MM,NN,OO^ | 1^KK^ | 2^LL,MM^ | 1^NN^ | 1^OO^ | 1^LL^ | 1^NN^ | 1^NN^ |
| T22 (17) | ***13*** \| 4 | 5 | 5/5 | 0/5 | 1/5 | ----- | AF-FISH (1)  AF-Karyo (1)  AF-Array (1)  AF-FISH + Array (1)  POC-Array (1) | 13 | 4^PP.QQ^ | 9 | 0 | 1^RR^ | 0 | 8^SS,TT,UU,^  ^VV,WW^ | 1^RR^ | 0 | 0 |

AF, amniotic fluid; Array, microarray; CB, cord blood; cFTS; combined first trimester screening; CV, chorionic villi; CVS, chorionic villus sampling; FGR, fetal growth restriction; IUFD, intrauterine fetal demise; Karyo, karyotype; LBW, low birth weight; M, monosomy; NBB, newborn blood; NICU, neonatal intensive care unit; PE, preeclampsia; PPROM, preterm premature rupture of membranes; SA, structural anomaly; T, trisomy; TAB, pregnancy termination; UC, umbilical cord; UPD, uniparental disomy; USS, ultrasound; VSD, ventral septal defect.

^A^Induced premature birth at 34/40 for severe IUGR (<5%ile). Neonate spent one month in NICU, doing well since..

^B^Absent flow through placenta prompting 29-week delivery, placenta 'grossly abnormal' on examination, NICU stay for neonate.

^C^Abnormal FTS (PAPP-A greatly decreased, hCG greatly increased, T21 risk for 1:1222); . IUGR and PPROM at 31/40. Spontaneous premature delivery at 34/40; low BW (below 5^th^ centile) but neonate well otherwise.

^D^ Normal parental karyotypes. IUGR and PPROM at 35/40, emergency caesarean section. Neonate had VSD and midline cystic structure in the brain.

^E^No obstetric details other than term, live-birth.­­

^F^Eight pregnancies with no reported complications and term delivery of a normal neonate.

^G^One pregnancy with no reported complications and term delivery of a normal neonate, ‘irritable uterus’, multiple admissions for preterm labour, induced for reduced fetal movement.

^H^One pregnancy was noted as progressing normally through 28 weeks and having presence of fibroid but no additional follow-up was provided. ^I^One pregnancy with oligohydramnios and IUGR, included preterm due to concerns for fetal wellbeing and slowing of growth velocity, birth weight <10%ile.
^J^One pregnancy spontaneous preterm birth.
^K^One pregnancy with spontaneous preterm birth and IUGR <5%ile.

^L^ One pregnancy spontaneous preterm birth, newborn had cephalohaematoma and was treated in hospital for jaundice (8 days). No other complications.

^M^IUFD at 13 weeks with no testing.

^N^Cleft lip/palate identified on ultrasound following NIPT; TAB

^O^One TAB due to mosaic T7 on diagnostic testing.

^P^TAB for single gene mutation unrelated to cfDNA findings.

^Q^One case with normal outcome also had normal maternal microarray.

^R^"Severe" IUGR and preterm birth (due to IUGR) in case with mosaic T8 on postnatal placental testing.
^S^TAB due to fetal anomalies; no testing or additional follow-up available.

^T^Miscarriage shortly after cfDNA screening.

^U^TAB after fetal confirmation of mosaic T10.

^V^IUFD at 14 weeks, POC after D&C confirmed T14 in all cells.

^W^Two cases with cotwin demise (one at 6 weeks, another at 7 weeks); repeat cfDNA screening at later GA was normal for both.
^X^One case with severe IUGR, TAB, anomalies on post-mortem exam, PGT-A tested mosaic embryo transfer (75% T15).
^Y^Three cases of 11 week IUFD.
^Z^One pregnancy with IUGR, term live-birth.
^AA^TAB in case with mosaicism and mUPD15 found on amniocentesis.

^BB^One case with 16p deletion on array, normal pregnancy outcome but child has developmental delays.

^CC^One case with cotwin demise at 6 weeks; repeat cfDNA screening at later GA had a signal below reportable level, likely due to demised twin. Ongoing twin normal, term live-birth.
^DD,EE^Two cases of preeclampsia with induced preterm delivery, one of which also had IUGR.
^FF^One case of emergency preterm delivery (C-section) due to preeclampsia at 34 weeks. Placenta appeared thick and slightly abnormal, mosaic T16 on placental testing.

^GG^Another preterm delivery had CPM for T16.
^HH^Preterm delivery was induced due to IUGR/fetal wellbeing with cleft palate noted on newborn exam.
^II^Two cases of IUFD with no testing—one at 13 weeks and another at 10-11 weeks.
^JJ^TAB in case with confirmed T16 mosaicism on amnio.

^KK^One case with pre-eclampsia, term delivery, live-born. ^LL^One case with IUGR, cord prolapse, emergency preterm delivery, 8 week NICU stay.
^MM^Case with spontaneous preterm birth.
^NN^TAB due to multiple anomalies on ultrasound, also seen on autopsy.
^OO^Fetal demise at 13 weeks, no testing.

^PP^Twin pregnancy, multiple anomalies in one baby and selective TOP prior to amnio, amnio on remaining twin WNL and normal birth outcome.
^QQ^Another case of twin demise at 6 weeks with normal repeat cfDNA screening.
^RR^One case with severe IUGR induced preterm.

^SS^ Two cases IUFD at 12 weeks, no testing.

^TT^ Three cases IUFD at 11 weeks, no testing.

^UU^IUFD at 12 weeks, cfDNA screen result confirmed on POC testing.

^VV^Spontaneous miscarriage at 13 weeks, no testing.

^WW^Spontaneous miscarriage at 12 weeks, no testing.
