# Supplementary figures and images for: Multisite assessment of the impact of cell-free DNA-based screening for rare autosomal aneuploidies on pregnancy management and outcomes
Source: Front Genet. 2022 Aug 29;13:975987. doi: 10.3389/fgene.2022.975987 (PMC9465083; doi:10.3389/fgene.2022.975987)

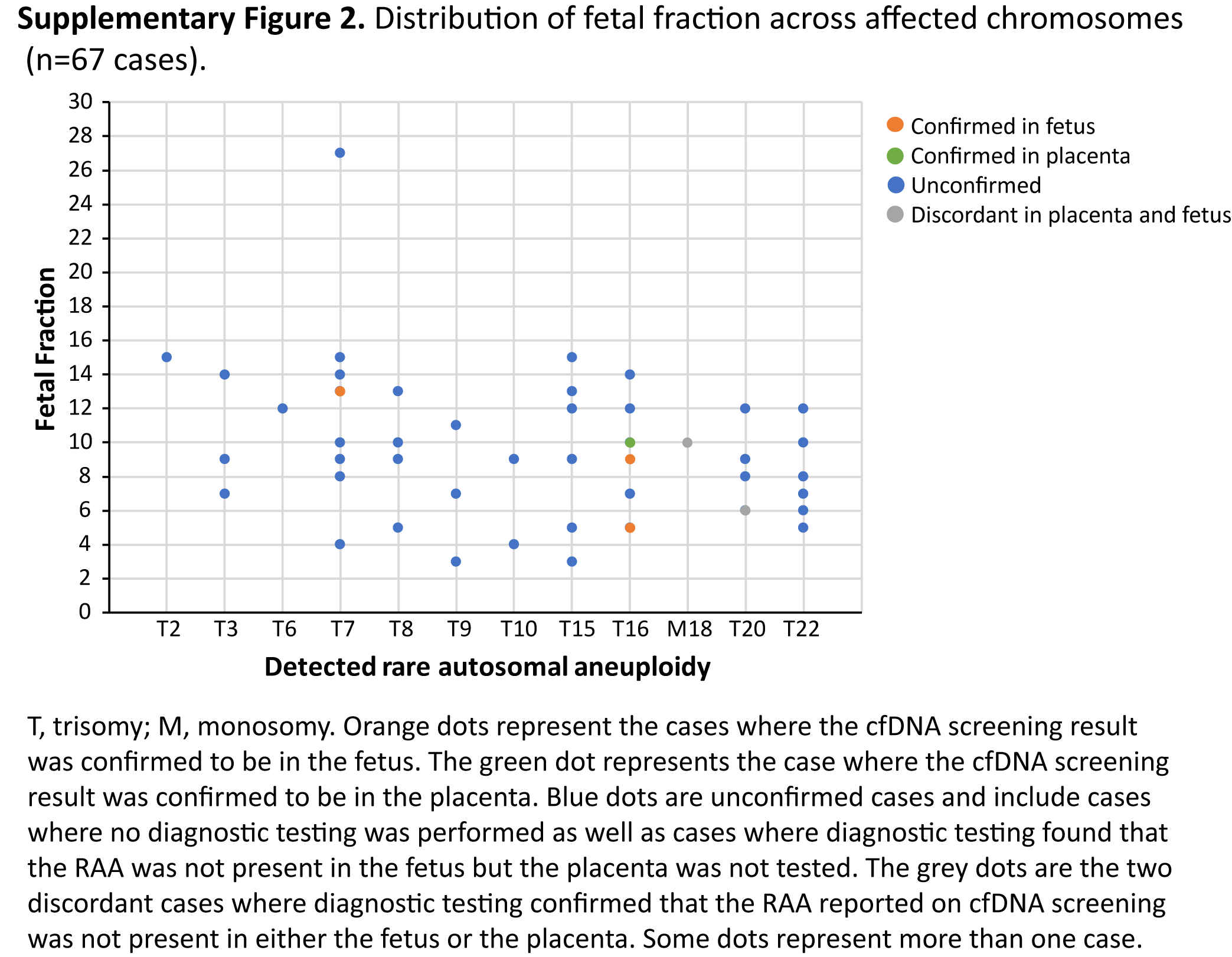

Supplement: Supplementary file 2 [file Image2.TIF]

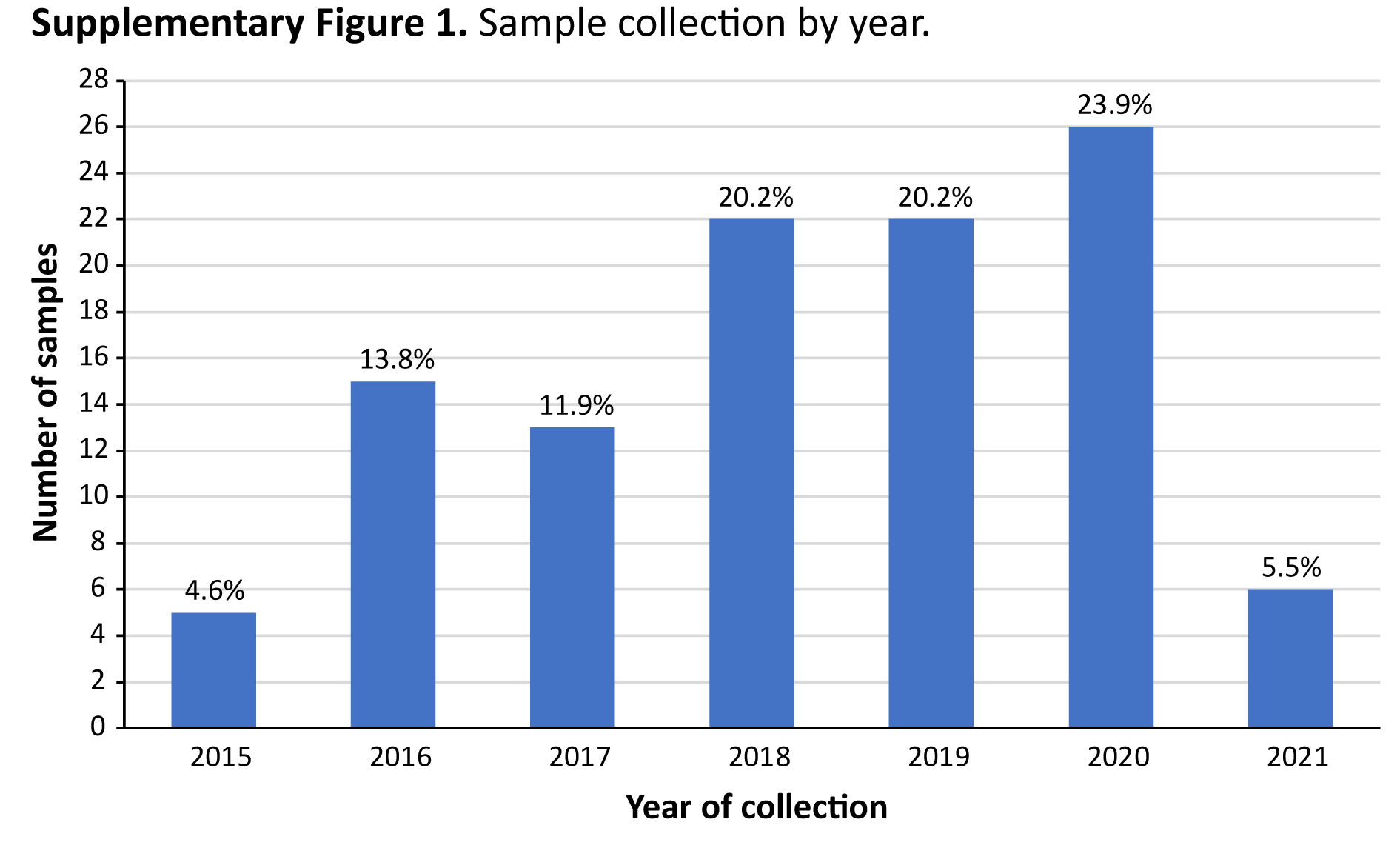

Supplement: Supplementary file 3 [file Image1.TIF]
