# Supplementary material for: Multisite assessment of the impact of cell-free DNA-based screening for rare autosomal aneuploidies on pregnancy management and outcomes
Source: Front Genet. 2022 Aug 29;13:975987. doi: 10.3389/fgene.2022.975987 (PMC9465083; doi:10.3389/fgene.2022.975987)
Supplement: Supplementary file 4 [file Table2.DOCX]

Supplementary Material

## Supplementary Table 2. Diagnostic testing carried out in patients.

| **Diagnostic Testing Performed** | **N (%)** |
| --- | --- |
| Yes  CVS  Amnio  Amnio/POC (cord)  Amnio/placental tissue (postnatal)  Amnio/POC (CV + UC)  Newborn testing (blood)  Newborn testing (blood)/ placental tissue (postnatal)  Umbilical cord/cord blood  Umbilical cord/placenta  POC | 74 (67.9)  2 (1.8)  59 (54.0)  1 (0.9)  2 (1.8)  1 (0.9)  3 (2.8)  1 (0.9)  1 (0.9)  2 (1.8)  2 (1.8) |
| No | 27 (24.8) |
| Unknown | 8 (7.3) |

Amnio, amniocentesis; CV, chorionic villi; CVS, chorionic villus sampling; POC, products of conception; UC, umbilical cord.
