# Supplementary material for: Multisite assessment of the impact of cell-free DNA-based screening for rare autosomal aneuploidies on pregnancy management and outcomes
Source: Front Genet. 2022 Aug 29;13:975987. doi: 10.3389/fgene.2022.975987 (PMC9465083; doi:10.3389/fgene.2022.975987)
Supplement: Supplementary file 5 [file Table3.DOCX]

Supplementary Material

## Supplementary Table 3. Results from Literature Review of Studies with RAAs Identified Through cfDNA Screening.

| **Publication** | **Population** | **Samples tested** | **RAA screen positives, n (%)** | **Outcomes reported** | **RAAs with outcomes** | **RAAs in fetus** | **CPM cases** | **UPD** | **Growth delay** | **SAB/IUFD no karyotype** | **Structural anomalies** | **Known cotwin demise** | **Maternal mosaicism confirmed** | **Normal birth outcome** |
| --- | --- | --- | --- | --- | --- | --- | --- | --- | --- | --- | --- | --- | --- | --- |
| (Ehrich et al., 2017) | High risk | 10, 272 | 80 (0.78) | None | 0 | ---- | ---- | ---- | ---- | ---- | ---- | ---- | ---- | ---- |
| (Lefkowitz et al., 2016) | High risk | 1,136^A^ | 8 (0.70) | Partial | 7 | 0 | 0 | UNK | UNK | UNK | UNK | UNK | UNK | UNK |
| (Pertile et al., 2017)  (Cohort 1) | Not specified | 72, 932 | 246 (0.34)^B^ | None | 0 | ---- | ---- | ---- | ---- | ---- | ---- | ---- | ---- | ---- |
| (Pertile et al., 2017)  (Cohort 2) | All risk | 16, 885 | 60 (0.36)^C^ | Partial | 52 | 6 | 5 | 7^D^ | 6 | 26 | 1 | 1 | 1 | ---- |
| (Pescia et al., 2017) | All risk | 6,388 | 50 (0.78) | Partial | 19 | 4 | UNK | 0 | UNK | UNK | UNK | UNK | UNK | UNK |
| (Oepkes et al., 2016) | High risk | 1,382^E^ | 15 (1.09) | Partial | 15 | 2 | UNK | UNK | UNK | UNK | 2 | UNK | UNK | UNK |
| (Zhu et al., 2020) | All risk | 437,873 | 247 (0.06) | Partial | 85 | 4 | 14 | 0 | 12 | 0 | 3 | UNK | UNK | UNK |
| (Zhu et al., 2021) | High risk | 943 | 133 (14.10) | Partial | 12 | 9 | 2 | UNK | UNK | UNK | UNK | UNK | UNK | UNK |
| (Phillips et al., 1996) | Not specified | 59,937 | 188 (0.31) | Partial | 6 | 6 | 182 | UNK | UNK | UNK | 35 | UNK | UNK | UNK |
| (Malvestiti et al., 2015) | Not specified | 60,347 | 542 (0.90) | Complete | 542 | 38 | 504 | 6^F^ | 2 | UNK | 3^G^ | 1^H^ | 0 | UNK |
| (Grati et al., 2006) | Not specified | 151,009 | 273 (0.18) | Partial | 203 | 0 | 203 | 1 | 1 | UNK | UNK | UNK | UNK | UNK |
| (Lau et al., 2014) | All risk | 1,959 | 9 (0.46) | Complete | 6 | 0 | 3 | 0 | 2 | 0 | 1 | UNK | 0 | UNK |
| (Liang et al., 2018) | All risk | 31,449 | 53 (0.17) | Partial | 49 | 0 | 0 | 3 | UNK | UNK | 3 | UNK | UNK | UNK |
| (Liang et al., 2019) | All risk | 94,085 | 49 (0.05) | Complete | 49 | 14 | UNK | 0 | UNK | UNK | UNK | UNK | UNK | UNK |
| (Xue et al., 2019) | All risk | 57,204 | 92 (0.16) | Partial | 43 | 1 | UNK | UNK | UNK | UNK | UNK | UNK | UNK | UNK |
| (Scott et al., 2018) | High risk | 23,388 | 28 (0.12) | Complete | 28 | 3 | 2 | 0 | 8 | 6 | 7^I^ | UNK | UNK | UNK |
| (Fiorentino et al., 2017) | All risk | 12,114 | 17 (0.14) | Complete | 17 | 10^J^ | NR | 1 | NR | 7 | NR | NR | NR | NR |
| (Van Den Bogaert et al., 2021) | All risk | 153,575 | 350 (0.23) | Partial | 272 | 11 | 28 | 3 | NR | NR | NR | NR | NR | NR |
| (Brison et al., 2018) | All risk | 19,735 | 58 (0.29) | Partial | 34 | 8 | ---- | ---- | ---- | ---- | ---- | ---- | ---- | ---- |
| (Colley et al., 2020) | All risk | 57^K^ | 10 (17.54) | Complete | 10 | 9 | NR | NR | UNK | 0 | UNK | n/a | NR | UNK |
| (He et al., 2019) | All risk | 42,924 | 72 (0.17) | Partial | 61 | 0 | 54 | 0 | 2 | NR | 0 | UNK | 0 | UNK |
| (Qi et al., 2019) | All risk | 31,250 | 35 (0.11) | Partial | 29^L^ | 0 | 10 | 0 | 3 | NR | 1^M^ | UNK | UNK | Majority |
| (Kleinfinger et al., 2020) | Mainly high risk | 3,007 | 25 (0.83) | None | ---- | ---- | ---- | ---- | ---- | ---- | ---- | ---- | ---- | ---- |
| (van der Meij et al., 2019) | All risk | 56, 818 | 101 (0.18) | Partial | 97 | 3 | 4 | 3 | NR | NR | NR | 0 | NR | NR |
| (Van Opstal et al., 2018) | High risk | 2,553 | 28 (1.10) | Partial | 27 | 4 | 22 | NR | 12 | NR | NR | 0 | NR | NR |
| (Soster et al., 2021) | All risk | 55,517 | 371 (0.67) | Partial | 183 | 41^N^ | NR | NR | NR | NR | NR | NR | NR | NR |

CPM, confined placental mosaicism; IUFD, intrauterine fetal demise; NR, not reported; RAA, rare autosomal aneuploidy; SAB, spontaneous abortion; UNK, unknown; UPD, uniparental disomy.

^A^Reportable samples.

^B^Does not include the 31 samples that had multiple abnormalities (mix of CNVs and aneuploidies).

^C^Does not include 3 cases with CNV in combination with trisomy.

^D^2/7 cases were putative/suspected UPD.

^E^Reported samples.

^F^True fetal mosaicism and confined placental mosaicism.

^G^At birth, the only two features resembling mosaic T8 were the presence of deep plantar furrows and one mal-positioned fourth toe on the left foot.

^H^Suspected co-twin demise.

^I^ Five fetal anomaly; Two liveborn with structural anomaly.

^J^Three had low level fetal mosaicism.

^K^Only reported samples with cytogenic results from products of conception analysis.

^L^25 patients underwent confirmatory diagnostic testing.

^M^This case had a 70.1-Mb duplication at 7q21.13-q36.3 combined with a 53.8-Mb deletion at Xp22.33-p11.22.

^N^True positives do not delineate fetal only vs placental only or both. Just defined as confirmed in fetus, placenta, or both.

**References**

Brison, N., Neofytou, M., Dehaspe, L., Bayindir, B., Van Den Bogaert, K., Dardour, L., et al. (2018). Predicting fetoplacental chromosomal mosaicism during non-invasive prenatal testing. *Prenat Diagn* 38(4)**,** 258-266. doi: 10.1002/pd.5223.

Colley, E., Devall, A.J., Williams, H., Hamilton, S., Smith, P., Morgan, N.V., et al. (2020). Cell-Free DNA in the Investigation of Miscarriage. *Journal of Clinical Medicine* 9(11)**,** 3428. doi: 10.3390/jcm9113428.

Ehrich, M., Tynan, J., Mazloom, A., Almasri, E., McCullough, R., Boomer, T., et al. (2017). Genome-wide cfDNA screening: clinical laboratory experience with the first 10,000 cases. *Genet Med* 19(12)**,** 1332-1337. doi: 10.1038/gim.2017.56.

Fiorentino, F., Bono, S., Pizzuti, F., Duca, S., Polverari, A., Faieta, M., et al. (2017). The clinical utility of genome-wide non invasive prenatal screening. *Prenat Diagn* 37(6)**,** 593-601. doi: 10.1002/pd.5053.

Grati, F.R., Grimi, B., Frascoli, G., Di Meco, A.M., Liuti, R., Milani, S., et al. (2006). Confirmation of mosaicism and uniparental disomy in amniocytes, after detection of mosaic chromosome abnormalities in chorionic villi. *Eur J Hum Genet* 14(3)**,** 282-288. doi: 10.1038/sj.ejhg.5201564.

He, Y., Liu, Y.H., Xie, R.G., Liu, S.A., and Li, D.Z. (2019). Rare autosomal trisomies on non-invasive prenatal testing: not as adverse as expected. *Ultrasound Obstet Gynecol* 54(6)**,** 838-839. doi: 10.1002/uog.20264.

Kleinfinger, P., Lohmann, L., Luscan, A., Trost, D., Bidat, L., Debarge, V., et al. (2020). Strategy for Use of Genome-Wide Non-Invasive Prenatal Testing for Rare Autosomal Aneuploidies and Unbalanced Structural Chromosomal Anomalies. *J Clin Med* 9(8). doi: 10.3390/jcm9082466.

Lau, T.K., Cheung, S.W., Lo, P.S., Pursley, A.N., Chan, M.K., Jiang, F., et al. (2014). Non-invasive prenatal testing for fetal chromosomal abnormalities by low-coverage whole-genome sequencing of maternal plasma DNA: review of 1982 consecutive cases in a single center. *Ultrasound Obstet Gynecol* 43(3)**,** 254-264. doi: 10.1002/uog.13277.

Lefkowitz, R.B., Tynan, J.A., Liu, T., Wu, Y., Mazloom, A.R., Almasri, E., et al. (2016). Clinical validation of a noninvasive prenatal test for genomewide detection of fetal copy number variants. *Am J Obstet Gynecol* 215(2)**,** 227.e221-227.e216. doi: 10.1016/j.ajog.2016.02.030.

Liang, D., Cram, D.S., Tan, H., Linpeng, S., Liu, Y., Sun, H., et al. (2019). Clinical utility of noninvasive prenatal screening for expanded chromosome disease syndromes. *Genet Med* 21(9)**,** 1998-2006. doi: 10.1038/s41436-019-0467-4.

Liang, D., Lin, Y., Qiao, F., Li, H., Wang, Y., Zhang, J., et al. (2018). Perinatal outcomes following cell-free DNA screening in >32 000 women: Clinical follow-up data from a single tertiary center. *Prenat Diagn* 38(10)**,** 755-764. doi: 10.1002/pd.5328.

Malvestiti, F., Agrati, C., Grimi, B., Pompilii, E., Izzi, C., Martinoni, L., et al. (2015). Interpreting mosaicism in chorionic villi: results of a monocentric series of 1001 mosaics in chorionic villi with follow-up amniocentesis. *Prenat Diagn* 35(11)**,** 1117-1127. doi: 10.1002/pd.4656.

Oepkes, D., Page-Christiaens, G.C., Bax, C.J., Bekker, M.N., Bilardo, C.M., Boon, E.M., et al. (2016). Trial by Dutch laboratories for evaluation of non-invasive prenatal testing. Part I-clinical impact. *Prenat Diagn* 36(12)**,** 1083-1090. doi: 10.1002/pd.4945.

Pertile, M.D., Halks-Miller, M., Flowers, N., Barbacioru, C., Kinnings, S.L., Vavrek, D., et al. (2017). Rare autosomal trisomies, revealed by maternal plasma DNA sequencing, suggest increased risk of feto-placental disease. *Sci Transl Med* 9(405). doi: 10.1126/scitranslmed.aan1240.

Pescia, G., Guex, N., Iseli, C., Brennan, L., Osteras, M., Xenarios, I., et al. (2017). Cell-free DNA testing of an extended range of chromosomal anomalies: clinical experience with 6,388 consecutive cases. *Genet Med* 19(2)**,** 169-175. doi: 10.1038/gim.2016.72.

Phillips, O.P., Tharapel, A.T., Lerner, J.L., Park, V.M., Wachtel, S.S., and Shulman, L.P. (1996). Risk of fetal mosaicism when placental mosaicism is diagnosed by chorionic villus sampling. *Am J Obstet Gynecol* 174(3)**,** 850-855. doi: 10.1016/s0002-9378(96)70312-5.

Qi, Y., Yang, J., Hou, Y., Guo, F., Peng, H., Wang, D., et al. (2019). The significance of trisomy 7 mosaicism in noninvasive prenatal screening. *Human Genomics* 13(1)**,** 18. doi: 10.1186/s40246-019-0201-y.

Scott, F., Bonifacio, M., Sandow, R., Ellis, K., Smet, M.E., and McLennan, A. (2018). Rare autosomal trisomies: Important and not so rare. *Prenat Diagn* 38(10)**,** 765-771. doi: 10.1002/pd.5325.

Soster, E., Boomer, T., Hicks, S., Caldwell, S., Dyr, B., Chibuk, J., et al. (2021). Three years of clinical experience with a genome-wide cfDNA screening test for aneuploidies and copy-number variants. *Genet Med* 23(7)**,** 1349-1355. doi: 10.1038/s41436-021-01135-8.

Van Den Bogaert, K., Lannoo, L., Brison, N., Gatinois, V., Baetens, M., Blaumeiser, B., et al. (2021). Outcome of publicly funded nationwide first-tier noninvasive prenatal screening. *Genetics in Medicine* 23(6)**,** 1-6. doi: 10.1038/s41436-021-01101-4.

van der Meij, K.R.M., Sistermans, E.A., Macville, M.V.E., Stevens, S.J.C., Bax, C.J., Bekker, M.N., et al. (2019). TRIDENT-2: National Implementation of Genome-wide Non-invasive Prenatal Testing as a First-Tier Screening Test in the Netherlands. *Am J Hum Genet* 105(6)**,** 1091-1101. doi: 10.1016/j.ajhg.2019.10.005.

Van Opstal, D., van Maarle, M.C., Lichtenbelt, K., Weiss, M.M., Schuring-Blom, H., Bhola, S.L., et al. (2018). Origin and clinical relevance of chromosomal aberrations other than the common trisomies detected by genome-wide NIPS: results of the TRIDENT study. *Genet Med* 20(5)**,** 480-485. doi: 10.1038/gim.2017.132.

Xue, Y., Zhao, G., Li, H., Zhang, Q., Lu, J., Yu, B., et al. (2019). Non-invasive prenatal testing to detect chromosome aneuploidies in 57,204 pregnancies. *Mol Cytogenet* 12**,** 29. doi: 10.1186/s13039-019-0441-5.

Zhu, X., Chen, M., Wang, H., Guo, Y., Chau, M.H.K., Yan, H., et al. (2021). Clinical utility of expanded non-invasive prenatal screening and chromosomal microarray analysis in high-risk pregnancy. *Ultrasound Obstet Gynecol* 57(3)**,** 459-465. doi: 10.1002/uog.22021.

Zhu, X., Lam, D.Y.M., Chau, M.H.K., Xue, S., Dai, P., Zhao, G., et al. (2020). Clinical Significance of Non-Invasive Prenatal Screening for Trisomy 7: Cohort Study and Literature Review. *Genes (Basel)* 12(1). doi: 10.3390/genes12010011.
