# Supplementary material for: Multisite assessment of the impact of cell-free DNA-based screening for rare autosomal aneuploidies on pregnancy management and outcomes
Source: Front Genet. 2022 Aug 29;13:975987. doi: 10.3389/fgene.2022.975987 (PMC9465083; doi:10.3389/fgene.2022.975987)
Supplement: Supplementary file 6 [file DataSheet1.DOCX]

Supplementary Material

**Appendix A.**

As noted in the Results section of the main manuscript, there were four cases that were listed as singleton pregnancies that had a demised twin prior to the first cfDNA screening blood draw. In the first case, there was a twin demise reported at seven weeks of gestation. Although the initial cfDNA screen carried out at 10.6 weeks’ gestation found trisomy 15, a repeat cfDNA screen at 14 weeks reported a normal result. No diagnostic testing was performed for this patient and the pregnancy resulted in a liveborn with no noted complications. The second case reported a twin demise at 6 weeks of gestation. The initial cfDNA screen noted a high-risk call for trisomy 15 at 11.1 weeks of gestation but a follow-up blood draw and analysis at 13.4 weeks was normal. Diagnostic testing and newborn physical exam were both normal. In the third case, a twin demise was also noted at 6 weeks of gestation. Cell-free DNA screening carried out at 10.7 weeks gestation found trisomy 22 but a repeat blood draw and analysis at 14.7 weeks noted that the trisomy 22 signal had dropped below a reportable level. No diagnostic testing was carried out, but the pregnancy resulted in a liveborn with no noted complications. The final case reported Trisomy 16 on cfDNA at 11 weeks following a twin demise at 6 weeks’ gestation. Sample recollection occurred at 13.4 weeks’ gestation and the Trisomy 16 signal dropped below the reportable cut-off value. There were no pregnancy complications and the surviving twin was born at term with no health issues noted.
